# A statistical model to identify determinants of glycemic control in patients with type 2 diabetes with different pharmacotherapeutic profiles

**DOI:** 10.1371/journal.pone.0235376

**Published:** 2020-07-06

**Authors:** Artur Mendes Moura, Marília Antunes, Sofia Oliveira Martins, João Filipe Raposo

**Affiliations:** 1 Department of Social Pharmacy, Faculty of Pharmacy, University of Lisbon, Lisboa, Portugal; 2 Department of Statistics and Operational Research, Faculty of Sciences, University of Lisbon, Lisboa, Portugal; 3 Centre of Statistics and its Applications of University of Lisbon (CEAUL), Lisboa, Portugal; 4 Department of Public Health, Nova Medical School, New University of Lisbon, Lisboa, Portugal; 5 Portuguese Diabetes Association (APDP), Lisboa, Portugal; Suez Canal University Faculty of Medicine, EGYPT

## Abstract

**Aim:**

To develop a statistical model to identify determinants of glycemic control.

**Materials and methods:**

A database was extracted from patients’ records with at least one glycated hemoglobin (HbA1c) analysis and with antidiabetic therapy established and stabilized. A logistic regression model was designed to identify the statistical significance of factors associated with glycemic control.

**Results:**

Higher probability of success (HbA1c ≤8% [64 mmol/mol]) was found for those who were older in age, those who were men, and those with higher education levels. Increased values for the following variables were associated with the poorest glycemic control: number of years of T2DM since diagnosis, number of antidiabetic medicines, body mass index, low-density lipoprotein cholesterol, triglycerides, systolic blood pressure and number of diabetes consultations in the last twelve months. The following pharmacotherapeutic treatments were associated with glycemic control (in decreasing order of the results): oral antidiabetic drugs; oral antidiabetic drugs and insulin; insulin. Patients using metformin and a dipeptidyl peptidase-4 inhibitors have a higher probability of success than do patients using metformin and a sulfonylurea, and patients using insulin and metformin have a higher probability of success than do patients using insulin alone.

**Conclusions:**

Sociodemographic, clinical and therapeutic parameters can strongly affect glycemic control. Studies based on real-life patient data provide important information on the development of more effective glycemic control.

## Introduction

Type 2 diabetes *mellitus* (T2DM) is a chronic disease whose onset can be a years-long process; moreover, T2DM can lead to multiple diabetes-related complications [1–3]. It is believed that good glycemic control is the best way to prevent diabetes complications [4]. However, 40–60% of individuals with T2DM are considered to have suboptimal glycemic control [5].

Patients with poor glycemic control who have multiple diabetes complications or who have had T2DM for many years are sometimes referred to specialized diabetes clinics, thus facilitating a multifactorial approach to this complex disease [6] and usually obtaining better health results [7]. A wide range of antidiabetic medicines is available [8], and several therapeutic schemes can be prescribed to patients, including treatment with one or more noninsulin drugs, insulin in monotherapy or a combination of these two types of medicines [9].

In some cases, after a patient’s clinical and nonclinical characteristics are analyzed, glycemic control may be considered to be acceptable even with a glycated hemoglobin (HbA1c) value above that generally recommended [10]. To achieve successful glycemic control, it is important to be aware of the related determinants [11].

The aim of this study was the creation of a statistical model based on logistic regression predicting the outcome of glycemic control from determinant variables, allowing for the adjustment of confounding effects in individuals with T2DM with stabilized antidiabetic pharmacotherapeutic treatment (oral antidiabetic drugs [OAD]; OAD and insulin; insulin). Other objectives of this study were the model re-estimations of the following treatments: i) metformin and a sulfonylurea *versus* metformin and a dipeptidyl peptidase-4 (DPP-4) inhibitor; and ii) insulin *versus* insulin and metformin.

## Materials and methods

The database was extracted from informatics records of a specialized clinic in diabetes (Portuguese Diabetes Association [APDP]). The database included the information of all patients who had at least one HbA1c laboratory analysis in 2012 (if patients had more than one analysis, the last one was used in the study) and who satisfied all the following criteria: i) at least one year of follow-up diabetes consultation; ii) at least one diabetes consultation in the last twelve months; and iii) antidiabetic therapy established and stabilized for at least 181 days.

The logistic regression model was designed with the objective of identifying statistically significant factors associated with glycemic control (HbA1c ≤8.0% [64 mmol/mol]). Age and sex were included in the model because they can be potential confounders. The education level variable was also included as a proxy for economic, social and health-related characteristics. This variable has some missing data (1694; 32.6%). However, it was included in the model because of its impact on the outcome. After these variables, other variables of the following groups were successively tested: i) diabetes characterization (age at time of T2DM diagnosis; years of T2DM since diagnosis); ii) antidiabetic pharmacological therapy (duration of current therapy; pharmacotherapeutic treatment [OAD; OAD and insulin; insulin]; number of different antidiabetic medicines); iii) laboratory parameters of renal function (glomerular filtration rate; albuminuria); iv) anthropometric, metabolic and behavioral characteristics (body mass index [BMI]; abdominal perimeter; total cholesterol; high-density lipoprotein [HDL] cholesterol; low-density lipoprotein [LDL] cholesterol; non-HDL cholesterol; triglycerides; both systolic and diastolic blood pressure [SBP and DBP]; smoking); and v) access to health care (medication reimbursement system; number of diabetes consultations in the last twelve months). In each group of variables, multicollinearity was evaluated to prevent the high correlation of variables in the model.

In the process of variable selection, the following aspects were considered: i) lower p-value associated with the significance of the parameter to the variable; ii) greater reduction in the value of the statistical Akaike information criterion; and iii) absence of collinearity problems with variables included in the previous stage model.

For statistical calculations, the software R (version 3.2.0) was used.

This study obtained approval from the Ethical Committee of APDP (number 273/2013; from 8th of April). The database was received in an anonymized information format.

## Results

### Statistical modeling of glycemic control (HbA1c ≤8.0% [64 mmol/mol]); antidiabetic pharmacotherapeutic treatment: OAD versus OAD and insulin versus insulin

As described in the Materials and Methods section, several variables were tested during the development of the logistic regression model of glycemic control (HbA1c ≤8.0% [64 mmol/mol]). Table 1 includes only variables that were statistically significant as variables non-statistically significant did not contribute to the statistical model.

**Table 1:** Summary of the statistical model for glycemic control: HbA1c ≤8.0% (64 mmol/mol); antidiabetic pharmacotherapeutic treatment: OAD *versus* OAD and insulin *versus* insulin.

Variable	Estimate	Standard error	z	^†^p-Value	^‡^OR	^‡^CI 95%; ^‡^OR
(Intercept)	2.147	0.515	4.168	<0.0001***		
Age (years)	0.019	0.004	4.409	<0.0001***	1.019	1.011–1.027
Sex:						
Woman (reference category)						
Man	0.240	0.077	3.121	0.0018**	1.271	1.093–1.478
Educational level:						
No education (reference category)						
Primary school	0.603	0.179	3.368	0.0008***	1.828	1.287–2.596
High school	0.726	0.193	3.761	0.0002***	2.067	1.416–3.017
Higher education	0.756	0.221	3.418	0.0006***	2.129	1.381–3.284
Years of T2DM since diagnostic	-0.010	0.004	-2.234	0.0255*	0.990	0.982–0.998
Pharmacotherapeutic treatment:						
OAD (reference category)						
OAD + Insulin	-0.807	0.096	-8.446	<0.0001***	0.446	0.370–0.539
Insulin	-1.235	0.114	-10.790	<0.0001***	0.291	0.233–0.364
Number of antidiabetic medicines	-0.223	0.042	-5.371	<0.0001***	0.800	0.737–0.869
BMI (kg/m^2^)	-0.021	0.008	-2.625	0.0087**	0.979	0.964–0.995
LDL-cholesterol (mg/dl)	-0.005	0.001	-4.643	<0.0001***	0.995	0.993–0.997
Triglycerides (mg/dl)	-0.002	<0.001	-3.955	0.0001***	0.998	0.998–0.998
SBP (mmHg)	-0.005	0.002	-2.703	0.0069**	0.995	0.991–0.999
Number of diabetes consultations in the last 12 months	-0.251	0.046	-5.477	<0.0001***	0.778	0.711–0.851

^†^Significance: • p-value- <0.10

*p-value <0.05

** p-value <0.01

*** p-value <0.001 /

^‡^ Odds ratio (OR) and Confidence interval (CI).

For model estimation, 3454 cases with complete information for the study variables were included. For more patient's details, please see Table 2 (column A).

**Table 2:** Patients’ main characteristics for model estimation with different pharmacotherapeutic profiles.

	A	B	C
Patients (N)	3454	284	1665
Age; average (years)	66.0	65.1	67.5
Sex			
Women (%)	49.0	42.3	53.6
Time since diagnosis of T2DM; average (years)	18.6	13.3	23.1
Pharmacotherapeutic treatment			
A OAD (n)	967		
OAD + Insulin (n)	1663		
Insulin (n)	824		
B Metformin + Sulphonylurea (n)		162	
Metformin + DPP-4 inhibitor (n)		122	
C Insulin (n)			824
Insulin + Metformin (n)			841
Glycaemic control			
Glycaemic controlled (n)	1777	213	759
Not glycaemic controlled (n)	1677	71	906

A (OAD / OAD + Insulin / Insulin); B (Metformin + Sulphonylurea / Metformin + DPP-4 inhibitor); C (Insulin / Insulin + Metformin).

Being older in age and being a man were significantly associated with better outcomes (Table 1). Additionally, having a higher level of education is progressively more associated with better outcomes. In contrast, a lower probability of success was associated with a longer length of time with T2DM, taking more antidiabetic medicines, and having higher values of BMI, LDL-cholesterol, triglycerides and SBP. More diabetes consultations are also associated with a lower probability of success. Patients taking OAD associated with insulin or taking insulin only have a significantly lower probability of success than do patients taking OAD.

### Model robustness

Only 44 cases (1.3%) were observed to be out of range for standardized Pearson residuals [-2;2]. Regarding the standardized deviance residuals, 7 cases (0.2%) were observed to be out of range. In relation to the diagonal values of matrix H (hat-values) and Cook distance values, only one case had high levels for both measures. This case also had a high standardized Pearson residual. This specific case had the following characteristics: adequate glycemic control (HbA1c ≤8.0% [64 mmol/mol]); 61 years old; man; high school education level; 17 years of T2DM since the diagnosis; OAD associated with insulin treatment; 3 antidiabetic medicines; BMI of 36.5 kg/m2; LDL-cholesterol of 117 mg/dl; triglyceride level of 1473.5 mg/dl; SBP of 145 mmHg; and 2 diabetes consultations in the last twelve months. The exclusion of this case and the re-estimation of the model did not change the meaning of the variable’s coefficients (Table 3). In this re-estimation, no cases showed high hat-values and high Cook's distance values simultaneously. Also, with respect to standardized Pearson residuals, there were no cases that were out of range.

**Table 3:** Estimate and odds ratio for recalculation of the statistical model for glycemic control (HbA1c ≤8.0% [64 mmol/mol]) after the exclusion of one specific case.

Variable	Estimate	^‡^OR
(Intercept)	2.149	
Age (years)	0.019	1.019
Sex:		
Woman (reference category)		
Man	0.246	1.278
Educational level:		
No education (reference category)		
Primary school	0.605	1.830
High school	0.727	2.068
Higher education	0.740	2.095
Years of T2DM since diagnostic	-0.010	0.990
Pharmacotherapeutic treatment:		
OAD (reference category)		
OAD + Insulin	-0.810	0.445
Insulin	-1.238	0.290
Number of antidiabetic medicines	-0.223	0.800
BMI (kg/m^2^)	-0.021	0.980
LDL-cholesterol (mg/dl)	-0.005	0.995
Triglycerides (mg/dl)	-0.002	0.998
SBP (mmHg)	-0.005	0.995
Number of diabetes consultations in the last 12 months	-0.250	0.779

^‡^ Odds ratio (OR).

To test the quality of the model adjustment, the Hosmer-Lemeshow test was performed. A total of G = 10 groups were considered, and a value of H = 8.071335 (p-value = 0.4265) was obtained; hence, evidence of a lack of fit was found. According to the McFadden proposal, the pseudo-R2 (0.3909) was calculated.

With this model, the area under the curve (AUC = 0.6942) for the receiver operating characteristic (ROC) curve was calculated. This value is in the higher borderline range of reasonable, which is very close to the range of good.

### Metformin and a sulfonylurea *versus* metformin and a DPP-4 inhibitor (HbA1c ≤8.0% [64 mmol/mol])

For comparison of different therapeutic treatments, the same model was applied by changing the pharmacotherapeutic treatment variable to the category metformin combined with a sulfonylurea and the category metformin combined with a DPP-4 inhibitor. In this model estimation, 284 cases were included. For more patient's details, please see Table 2 (column B).

The dummy variable for the category metformin and a DPP-4 inhibitor presents a positive value for the coefficient estimate and a p-value <0.01 (Table 4). These results indicate that, maintaining the conditions for the other variables, patients with metformin and a DPP-4 inhibitor treatment have a higher probability of success (HbA1c ≤8.0% [64 mmol/mol]), with an increase of 175.3% [CI 95% 1.325;5.721], than patients with metformin and a sulfonylurea treatment. Both high school and higher education levels also have positive coefficient estimates and are statistically significant.

**Table 4:** Summary of the statistical model for glycemic control (HbA1c ≤8.0% [64 mmol/mol]) in patients on metformin and a sulfonylurea treatment *versus* in patients on metformin and a DPP-4 inhibitor treatment.

Variable	Estimate	Standard error	z	^†^p-Value	^‡^OR	^‡^CI 95%; ^‡^OR
(Intercept)	-1.384	2.377	-0.582	0.560		
Age (years)	0.028	0.018	1.529	0.126	1.028	0.992–1.066
Sex:						
Woman (reference category)						
Man	0.563	0.316	1.780	0.075•	1.756	0.945–3.266
Educational level:						
No education (reference category)						
Primary school	1.536	0.800	1.921	0.055•	4.648	0.969–22.289
High school	2.229	0.858	2.597	0.009**	9.292	1.728–49.973
Higher education	2.062	0.913	2.259	0.024*	7.865	1.314–47.078
Years of T2DM since diagnostic	-0.001	0.020	-0.070	0.944	0.999	0.960–1.039
Number of antidiabetic medicines	-0.293	0.333	-0.880	0.379	0.746	0.388–1.434
BMI (kg/m^2^)	-0.007	0.033	-0.217	0.828	0.993	0.931–1.059
LDL-cholesterol (mg/dl)	-0.003	0.005	-0.572	0.567	0.997	0.988–1.007
Triglycerides (mg/dl)	0.000	0.002	-0.212	0.832	1.000	0.996–1.003
SBP (mmHg)	-0.006	0.007	-0.780	0.435	0.994	0.980–1.009
Number of diabetes consultations in the last 12 months	0.196	0.242	0.810	0.418	1.216	0.757–1.953
Pharmacotherapeutic treatment:						
Metformin + Sulfonylurea (reference category)						
Metformin + DPP-4-inhibitor	1.013	0.373	2.714	0.007**	2.753	1.325–5.721

^†^Significance:

• p-value <0.10

* p-value <0.05

** p-value <0.01

*** p-value <0.001 /

^‡^ Odds ratio (OR) and Confidence interval (CI).

### Insulin *versus* insulin and metformin (HbA1c ≤8.0% [64 mmol/mol])

For the comparison of insulin therapy *versus* insulin and metformin therapy, the same model was applied by changing the respective categories of pharmacotherapeutic treatment variables. In this model estimation, 1665 cases were included. For more patient's details, please see Table 2 (column C).

The dummy variable for the category insulin and metformin treatment presents a positive value for the coefficient estimate and a p-value <0.01 (Table 5). These findings indicate that, maintaining the conditions for the other variables, patients with insulin and metformin treatment have a higher probability of success (HbA1c ≤8.0% [64 mmol/mol]), with an increase of 71.8% [CI 95% 1.329;2.220], than the patients with insulin monotherapy treatment. Age and sex (men) have positive coefficient estimates and are statistically significant. The number of antidiabetic medicines, BMI, LDL-cholesterol, triglycerides and the number of diabetes consultations in the last twelve months have negative coefficient estimates and are statistically significant.

**Table 5:** Summary of the statistical model for glycemic control (HbA1c ≤8.0% [64 mmol/mol]) in patients on insulin treatment *versus* in patients on insulin and metformin treatment.

Variable	Estimate	Standard error	z	^†^p-Value	^‡^OR	^‡^CI 95%; ^‡^OR
(Intercept)		0.741	1.909	0.056•		
Age (years)	0.018	0.006	2.930	0.003**	1.018	1.006–1.031
Sex:						
Woman (reference category)						
Man	0.310	0.109	2.839	0.005**	1.364	1.101–1.689
Educational level:						
No education (reference category)						
Primary school	0.275	0.236	1.161	0.246	1.316	0.828–2.092
High school	0.396	0.260	1.521	0.128	1.486	0.892–2.475
Higher education	0.485	0.329	1.474	0.141	1.624	0.852–3.095
Years of T2DM since diagnostic	-0.007	0.006	-1.116	0.264	0.993	0.982–1.005
Number of antidiabetic medicines	-0.321	0.085	-3.785	<0.001***	0.725	0.614–0.856
BMI (kg/m^2^)	-0.024	0.011	-2.129	0.033*	0.976	0.955–0.998
LDL-cholesterol (mg/dl)	-0.005	0.002	-3.273	0.001**	0.995	0.991–0.998
Triglycerides (mg/dl)	-0.001	<0.001	-2.270	0.023*	0.999	0.998–1.000
SBP (mmHg)	-0.005	0.003	-1.794	0.073•	0.995	0.991–1.000
Number of diabetes consultations in the last 12 months	-0.335	0.066	-5.070	<0.001***	0.715	0.629–0.814
Pharmacotherapeutic treatment:						
Insulin (reference category)						
Insulin + Metformin	0.541	0.131	4.132	<0.001***	1.718	1.329–2.220

^†^Significance:

• p-value <0.10

* p-value <0.05

** p-value <0.01

*** p-value <0.001 /

^‡^ Odds ratio (OR) and Confidence interval (CI).

## Discussion

There are several studies already published related with determinants of glycemic control but there are different values to consider that glycemic control is achieved. Concerning the present study, it is adequate to start the discussion with results from studies that chose approximately the same target value.

In a nationwide study in the United States of America, with the outcome of success defined as HbA1c <8.0% (64 mmol/mol), a logistic regression established statistical significance for the variables age (older), sex (men) and therapeutic profile; furthermore, the probability of success for therapeutic profile was as follows (in ascending order): OAD conjugated with insulin; only insulin; only OAD [12]. Another nationwide epidemiologic study in Brazil, which also defined the outcome of success as HbA1c <8.0% (64 mmol/mol), found a higher probability of success in older people, patients who had T2DM for a shorter period of time and patients who were not under insulin treatment; these findings were statistically significant [13]. In general, these studies are in line with the determinants found on the present study; there is a higher probability of glycemic control for men, older patients, and patients with less years of T2DM since diagnosis. A common finding is that patients taking insulin have a lower probability of achieving glycemic control. Usually patients are first treated with OAD being insulin added to treatment at a later stage when the glycemic control fails. Thus, it is reasonable that insulin is associated with the poorest results for glycemic control.

Other findings of the present study are that an increased number of antidiabetic medicines prescribed and an increased number of diabetes consultations are associated with a worst glycemic control, what probably reflects the fact that patients with poor glycemic control need more drugs or tighter clinical follow-up.

Despite other studies selected lower glycemic control values, those studies are important to have an overview of their findings. One study in primary care units in the United Kingdom used logistic regression to examine glycemic control, defined as HbA1c ≤7.5% (59 mmol/mol), and revealed a higher probability of success in older people and in patients treated with five or fewer medicines (not only antidiabetic medicines); these findings were statistically significant [14]. For glycemic control defined as HbA1c ≤7.0% (53 mmol/mol), another study in Brazil performed in ambulatory patients from an university clinic detected a higher probability of success in older people, patients with ≤100 mg/dl of LDL-cholesterol and patients without insulin treatment; these findings were statistically significant [15]. For the same glycemic control level, a study performed in an endocrinology clinic in Beirut, Lebanon, showed a higher probability of success in patients with <150 mg/dl of triglycerides and without insulin treatment; these findings were statistically significant [16]. A study of glycemic control, defined as HbA1c <7.0% (53 mmol/mol), that was performed in nationwide Chinese hospitals found a higher probability of success in older people, women and those only under OAD treatment (instead of OAD combined with insulin treatment); these findings were statistically significant [17]. In individuals from the Jordan National Center for Diabetes, there was a higher probability of success in patients who had T2DM for seven or fewer years; with respect to the therapeutic profile, the ascending probability of success was as follows: both OAD and insulin; only insulin; only OAD [18].

In these studies, the statistically significant variables are in line with the present study, except for the study performed in Chinese in hospitals where women got more probability of glycemic control. In the present study many metabolic variables where statistically significant (BMI, LDL-cholesterol, triglycerides and SBP) and only two studies [15, 16] obtained statistical significance for this type of variables.

However, not only clinical parameters are important to glycemic control. One of the most important finding in the present study was that educational level has a strong association to a successful glycemic control. This variable was not analysed or relevant in the studies previously mentioned.

Concerning the specific treatments of metformin combined with a sulfonylurea *versus* metformin combined with a DPP-4 inhibitor, one German multicenter study followed individuals with these treatments for twelve months. For the first treatment, at the beginning of the study, patients had T2DM for an average of 5.4 years and had an average HbA1c value of 7.6±0.8% (60±9 mmol/mol); at the end of the study, an average change of -0.6±0.9% (-7±10 mmol/mol) was observed. For the second treatment, at the beginning of the study, patients had T2DM for an average of 4.7 years and an average HbA1c value of 7.5±0.7% (59±8 mmol/mol); at the of the study, an average change of -0.6±0.8% (-7±9 mmol/mol) was observed. No statistical significance was observed between these two average changes in HbA1c [19].

In one study that focused on the addition of a sulfonylurea or a DPP-4 inhibitor to metformin, the use of a DPP-4 inhibitor led to a higher proportion of patients with good glycemic control (HbA1c <7% [53 mmol/mol]); these findings were statistically significant [20]. Another study also showed better results with a DPP-4 inhibitor than with a sulfonylurea, both in addition to metformin, but these findings did not achieve statistical significance [21]. On the present study, concerning the addition of a DDP-4 inhibitor *versus* a sulfonylurea to metformin, the result is very clear, as a strong statistical association was obtained: adding a DDP-4 inhibitor leads to a better probability of glycemic control. However, we need to consider that different populations, settings, and methodologies can lead to different outcomes. Regardless the medicine added to metformin treatment, the introduction of a second medicine, namely, a sulfonylurea or a DPP-4 inhibitor, leads to a decrease in HbA1c levels [20–24].

One meta-analysis that included twenty studies on treatment with both insulin and metformin *versus* treatment with only insulin presented an average reduction in HbA1c of 0.6% (7 mmol/mol) (CI 95% [-0.89, -0.31], p<0.001) in those treated with insulin combined with metformin [25]. The reduction in HbA1c levels with this type of drug combination continues to be described in more recent studies [26, 27] and they are in line with the strong statistic association revealed in the present study.

Regarding the limitations of this study, the information on this database is from 2012, and we now have additional medicines. However, this advancement does not invalidate the statistical model and the comparison of different therapeutic treatments. The database was extracted from clinic records and these records do not have information related with important aspects such as income, rural or non-rural residence, marital status, and if the patient leaves alone or not. Other important clinic information, such as other diseases or patient's phenotypes could not consistently be extracted from the records.

With respect to the setting (APDP), this clinic is established in Lisbon and it is well known in Portugal. Many patients with diabetes are referred to this clinic, especially patients without glycemic control, and all patients are follow-up by their own diabetologist doctor. The statistical model was created for the outcome HbA1c ≤8.0% (64 mmol/mol) because in the population under study, this glycemic level is a realistic goal with respect to patients’ profiles [10].

In Portugal anyone living in the country has access to National Health Service (NHS). This clinic has a protocol with the NHS, so it receives all kind of people, irrespectively of their origin and level income. However, because of its location, most patients came from Lisbon Metropolitan Area. Therefore, this clinic is not representative of the Portuguese population with diabetes, but it supports many patients with a difficult glycemic control.

## Conclusions

In patients with T2DM, the determinants of the achievement of glycemic control are very important. In this study, being older in age, being a man, and having a higher education level were associated with better glycemic control. The increase in the value of the following variables was associated with the poorest glycemic control: number of years since T2DM diagnosis, number of antidiabetic medicines, BMI, LDL-cholesterol, triglycerides, SBP and number of diabetes consultations in the last twelve months.

The pharmacotherapeutic treatment is associated with glycemic control. The patients treated with OAD have more probability of glycemic control than patients treated with OAD and insulin. The patients taking only insulin have the poorest probability of glycemic control.

When glycemic control is not established with metformin treatment, it is crucial to choose a second medicine. The addition of a DPP-4 inhibitor or a sulfonylurea should be contemplated, with better results achieved with the former.

In patients with insulin treatment, maintaining metformin can be useful for better glycemic control.

Finally, to develop better glycemic control, it is very important to understand the information provided by studies based on real-life patient data.

## Supporting information

S1 Data (SAV)
